# Chronic Aseptic Myometritis: A Mechanistic Framework Linking Sterile Myometrial Inflammation to Uterine Fibroid Initiation and a Roadmap for Primary Prevention

**DOI:** 10.3390/cells15161469

**Published:** 2026-08-17

**Authors:** Saba Haq, Fatimah Hussein, Ola Elamin, Mervat M. Omran, Jakub Kociuba, Michal Ciebiera, Mahya Mohammadi, Esra Cetin, Everett Tate, Obianuju Sandra Madueke-Laveaux, Mira Mousa, Mostafa Borahay, Mohamed Ali, Ayman Al-Hendy

**Affiliations:** 1 Department of Medical Science, Khalifa University, Abu Dhabi 127788, United Arab Emirates; saba.ahaq@ku.ac.ae (S.H.); 100059408@ku.ac.ae (F.H.); 100066983@ku.ac.ae (O.E.); 2 Department of Obstetrics and Gynecology, University of Chicago, Chicago, IL 60637, USAetate@bsd.uchicago.edu (E.T.); mohamed.ali@bsd.uchicago.edu (M.A.); 3 Cancer Biology Department, National Cancer Institute, Cairo University, Cairo 11769, Egypt; 4 Department of Gynecology, Obstetrics and Gynecological Endocrinology, 00-189 Warsaw, Poland; 5 Warsaw Institute of Women’s Health, 00-189 Warsaw, Poland; 6 Development and Research Center of Non-Invasive Therapies, Pro-Familia Hospital, 35-302 Rzeszow, Poland; 7 Hurley Medical Center, Michigan State University, East Lansing, MI 48503, USA; 8 Department of Public Health and Epidemiology, College of Health and Medical Sciences, Khalifa University, Abu Dhabi 127788, United Arab Emirates; 9 Center for Biotechnology, College of Health and Medical Sciences, Khalifa University, Abu Dhabi 127788, United Arab Emirates; 10 Department of Gynecology & Obstetrics, Johns Hopkins University, Baltimore, MD 21287, USA; mboraha1@jhmi.edu; 11 Clinical Pharmacy Department, Faculty of Pharmacy, Ain Shams University, Cairo 11566, Egypt

**Keywords:** uterine fibroids, chronic aseptic myometritis, abnormal uterine bleeding, sterile inflammation, DAMPs, NLRP3 inflammasome, oxidative DNA damage, extracellular matrix remodeling, TGF-β signaling

## Abstract

Uterine fibroids, the most common tumors in reproductive-age women, remain without a defined precursor tissue state. Unlike cervical dysplasia preceding cervical cancer, or colonic polyps preceding colorectal malignancy, no equivalent “at-risk” tissue marker exists for fibroids, and diagnosis relies on radiological imaging only after tumors are already well-established and often symptomatic including excessive menstrual bleeding, pelvic pain, infertility and obstetric complications. In this narrative review, we propose that a subset of women with unexplained AUB may harbor a chronic, non-infectious inflammatory condition of the myometrium, which we term Chronic Aseptic Myometritis (CAM). We synthesize mechanistic and human tissue evidence suggesting that sterile inflammation driven by damage-associated molecular patterns, NLRP3 inflammasome activation, oxidative DNA damage, and TGF-β–mediated extracellular-matrix remodeling may underlie the transition from normal myometrium (MyoN) to a pre-fibroid, inflamed and stiffened state (MyoF), and may contribute both to abnormal uterine bleeding (AUB) and to fibroid initiation. We propose a preliminary framework for future CAM research, including the identification of candidate biomarker categories and imaging correlates. We also discuss whether early mechanism-based interventions, such as vitamin D and epigallocatechin gallate (EGCG), may offer a potential pathway toward primary prevention. Because the components of this model derive largely from experimental and cross-sectional human studies, CAM is presented as a hypothesis-generating, myometrium-centered framework rather than a validated clinical entity, and prospective validation is required.

## 1. Introduction

Uterine fibroids (UFs, also known as leiomyomas) are the most common benign tumors in women of reproductive age, arising from the clonal expansion of a single mutated abnormal myometrial stem cell. UFs cause gynecologic and reproduction conditions including prolonged menstrual bleeding, pelvic pain and anemia, recurrent pregnancy loss, subfertility, and preterm labor [1,2]. These complications impart substantial economic burden, and the annual expenditure of UF care exceeds USD 42 billion per year [3]. Currently, there are no FDA-approved drugs used for the long-term treatment of UFs. In contrast, in Europe, Relugolix combination therapy and Linzagolix with add-back therapy are approved for sustained management of UF-associated heavy menstrual bleeding in appropriately selected patients [4,5,6]. Although surgery remains the mainstay, myomectomy and hysterectomy carry peri-operative (hemorrhage, infection, vital organ injury) and long-term risks (adhesions, recurrence, loss of fertility, heart and metabolic complications along with dementia in hysterectomy cases), underscoring the need to pivot from reactive procedures to proactive prevention [7,8,9]. Mechanism-based research into UF pathogenesis is therefore essential for risk stratification, early intervention, and most importantly, primary prevention.

Chronic inflammation usually starts as a low-grade, persistent response to cellular stress, tissue injury, and hormonal imbalance. In the presence of a tightly regulated process, the inflammatory response resolves rapidly, and normal tissue architecture is restored. However, if inflammation becomes chronic, it may result in pathological fibrosis and impair normal tissue function [10,11,12,13]. A normal cell can become tumorous after repeated DNA instability secondary to chronic inflammation, which triggers tumor-initiating genetic events through damaged DNA repair mechanisms. Mechanistically, chronic inflammation leads to reactive oxygen and nitrogen species (ROS/RNS) generation that oxidize genomic DNA, produce 8-hydroxy-2′-deoxyguanosine (8-OHdG) while simultaneously impairing DNA repair. This results in the acquisition of fibroid driver mutations in myometrial stem cells [14,15,16,17]. Three main fibroid driver alterations have been identified, including MED12 mutations (around 70–80% of leiomyomas), HMGA2-driven tumors (around 10–15%), and rarer fumarate hydratase (FH)-deficient leiomyomas (approximately 1%) [18,19,20]. MED12 mutation disturbs the CDK8 kinase module of the Mediator complex and abnormally activates Wnt4/β-catenin signaling [19,21]; HMGA2 overexpression arises from 12q14 chromosomal rearrangement and induces pro-proliferative transcription [20]; and FH deficiency leads to oncometabolite fumarate accumulation which contributes to pseudohypoxia and oxidative stress [18,20]. These driver mutations transform inflamed myometrial stem cells into fibroid-producing stem cells [22,23,24,25]. These fibroid-forming stem cells mainly differentiate into fibroid tumor cells which produce excessive extracellular matrix (ECM) and demonstrate heightened cellular proliferation and apoptosis inhibition, leading to fibroid development [16,26].

### 1.1. From Normal Myometrium to Pre-Fibroid Myometrium: The MyoN–MyoF Transition

**Figure 1:**
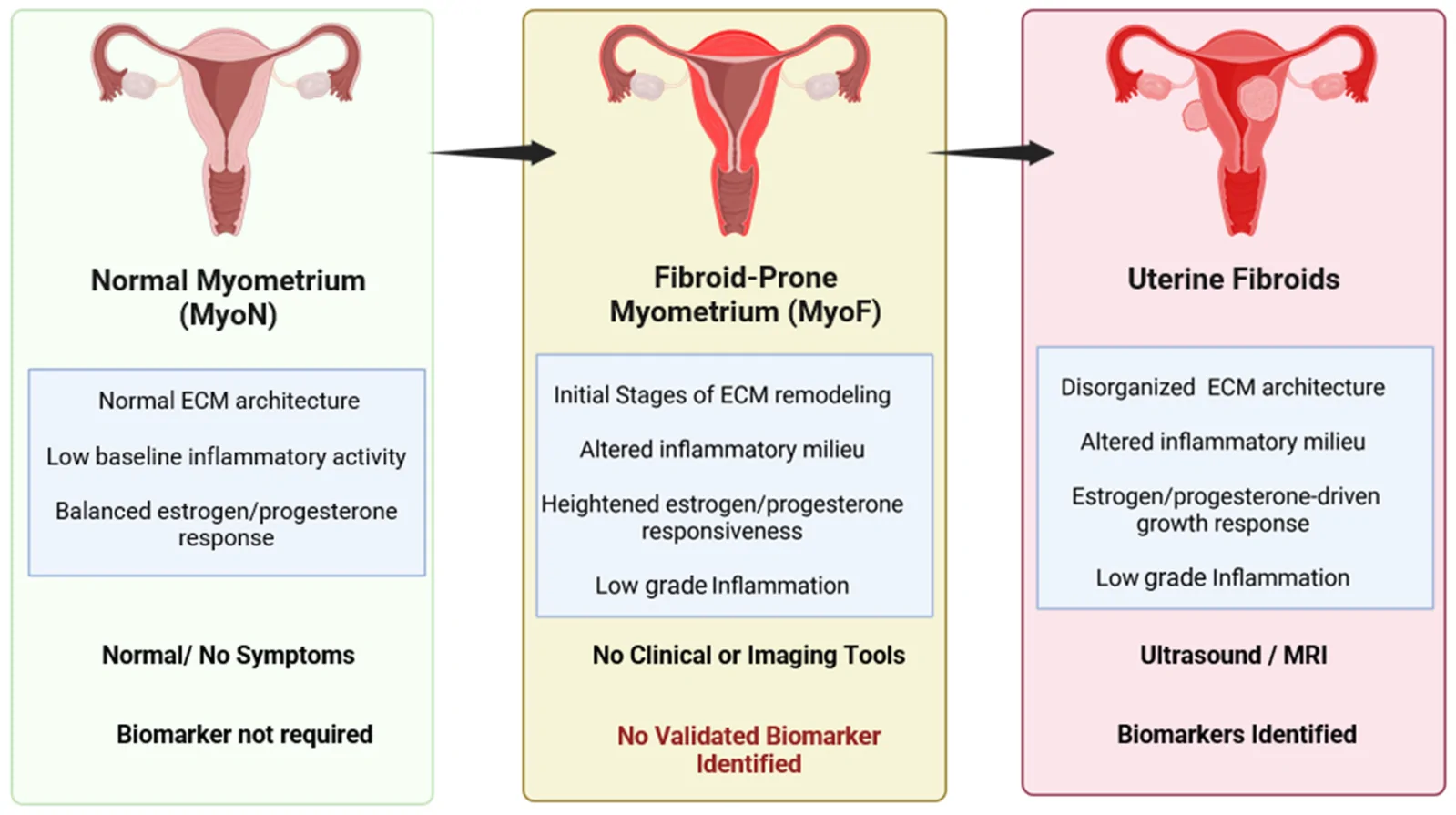
Proposed progression from normal myometrium (MyoN) to pre-fibroid myometrium (MyoF) and clinically detectable uterine fibroids. MyoN is characterized by organized extracellular matrix (ECM), low baseline inflammatory activity, and physiologic hormonal responsiveness. MyoF is proposed as a fibroid-prone state marked by early ECM remodeling, low-grade inflammation, and altered steroid-hormone responsiveness, but currently lacks validated biomarkers or routine diagnostic imaging tools. With continued remodeling and inflammatory dysregulation, this state may progress to clinically detectable uterine fibroids, which exhibit disorganized ECM architecture, growth responsiveness, and established imaging detectability. Figure created with https://www.biorender.com/.

Our recent studies clearly demonstrate a key role of myometrial inflammation as a prerequisite for subsequent uterine fibroids development [14,20,27]. The myometrium adjacent to fibroids (MyoF) is biologically distinct from fibroid-free myometrium (MyoN). The healthy, fibroid-free myometrium (MyoN) can transition to a pre-fibroid myometrium (MyoF) defined by inflammatory signaling and ECM–mechanical remodeling [15,21,28,29]. This proposed intermediate state may precede UF development, as unresolved inflammation drives ECM deposition and tissue remodeling could result in persistent structural and functional impairment of the myometrium (Figure 1).

This distinction between MyoN and MyoF is supported by accumulating molecular and functional evidence. For instance, Sharan et al. compared MyoN with MyoF and showed differential expression of chromatin-modifying enzymes, proposing epigenomic dysregulation in fibroid biology [30]. Prusinski Fernung et al. demonstrated that fibroid Stro-1^+^/CD44^+^ stem cells exhibit increased DNA damage and impaired DNA repair compared with adjacent myometrial stem cells, supporting that genomic instability in MyoF stem cells facilitates acquisition of driver mutations such as MED12 [31]. Paul et al. showed that MyoF is transcriptomically distinct from MyoN, with enrichment of inflammatory and ECM-remodeling pathways, establishing MyoF as a pre-fibroid state [32]. Methylome and transcriptome analyses showed race-specific differences in MyoF biology, as Black women myometrium showed greater expression of fibroid-related genes. This suggested that myometrium is susceptible to fibroid development prior to tumor formation [33]. Li et al. linked a stressed MyoF microenvironment to mutagenesis by demonstrating that myometrial oxidative stress promotes MED12 mutations through 8-OHdG-mediated mis-repair, thereby connecting reactive oxygen species (ROS)-damaged myometrial stem cells to fibroid-initiating mutations [17]. In parallel, Bariani et al. reported that MyoF from susceptible Black women is enriched for ECM, mechano-sensing, and p38 MAPK signaling pathways, supporting that MyoF is inflamed, stiff, and mechanically changed [27]. Consistent with these findings, human myometrial and fibroid stem cell-derived 3D organoids exhibit exaggerated ECM deposition and heightened stiffness in fibroid-derived organoids compared with normal myometrial organoids [34,35]. Most recently, Paul et al. showed that the myometrial transcriptome and methylome of testosterone can push healthy myometrium toward a MyoF-like, fibroid-prone state [36]. Together, these data demonstrate that MyoF significantly differs from MyoN, with altered gene expression, increased oxidized DNA, and heightened responses to ROS.

Mechanistically, such alterations may converge on transforming growth factor-β3 (TGF-β3) as a central profibrotic node. TGF-β3 activates Smad2/3 signaling, resulting in collagen and fibronectin synthesis and ECM accumulation, while also suppressing nucleotide-excision DNA repair. This links fibrotic remodeling to genomic instability and driver-mutation acquisition [14,20,26,27]. Matrix stiffening is not confined to mere structural elements; rather, it actively signals through pathways including p38 MAPK, YAP/TAZ (Hippo), and the mechanically gated channel PIEZO1 to promote pro-fibrotic and pro-proliferative gene expression, thereby promoting additional ECM deposition and stiffening [26,37,38]. Taken together, such processes establish a self-reinforcing mechano-inflammatory loop that may describe the reason for which MyoF state, once initiated, persists and progresses rather than resolves.

### 1.2. Abnormal Uterine Bleeding and the FIGO PALM-COEIN Framework

Abnormal uterine bleeding (AUB) is one of the most common gynecologic complaints in adolescents and reproductive-age women and often serves as the earliest clinical manifestation of underlying uterine dysfunction. In the context of this review, AUB is particularly relevant because a substantial subset of cases remains unexplained after routine clinical evaluation, raising the possibility that occult myometrial pathology may contribute to their origin. The complexity of AUB’s etiology requires a comprehensive classification system. The FIGO classification, recognized as the most current and widely adopted system, categorizes AUB causes under the PALM-COEIN acronym, delineating the most common origins [39,40]. In cases where no anatomical or systemic diseases are identified, we categorize AUB into AUB-O (indicative of ovulatory dysfunction), AUB-E (suggesting non-anatomical endometrial disorders), and AUB-N, where the cause remains unidentified [39]. This classification modernizes and covers the concept previously known as dysfunctional uterine bleeding (DUB), which was a diagnosis of exclusion in case of AUB [39,41].

Although dysfunctional uterine bleeding (DUB), recently incorporated within the FIGO AUB framework, can occur at any age, it is particularly common in adolescents and perimenopausal women because of the higher frequency of anovulatory cycles in these groups [39,41,42]. In adolescents, irregular or heavy bleeding during the first few years after menarche often reflects immaturity of the hypothalamic–pituitary–ovarian axis and the resulting lack of consistent ovulation [43,44,45]. This can make it difficult to distinguish physiologic menstrual irregularity from pathologic bleeding, especially because DUB remains a diagnosis of exclusion and normal menstrual patterns may not yet be established in this age group [45]. In anovulatory cycles, persistent estrogen exposure without adequate progesterone-mediated stabilization may promote excessive endometrial proliferation and irregular shedding, contributing to prolonged or heavy bleeding [45,46].

Abnormal bleeding in the absence of structural or systemic disease is not confined to anovulatory patients. It can also occur in regularly ovulating women of reproductive age, in whom menstrual bleeding is often regular but excessive [47]. In these women, endometrial molecular abnormalities have been proposed, including imbalance between vasoconstrictors such as endothelin-1 and prostaglandin F2alpha and vasodilators such as prostaglandin E2 and prostacyclin I2, as well as increased fibrinolysis through enhanced plasminogen activator production [39,45]. The possible influence of endometrial inflammation is considered as the primary trigger of these local hormonal changes. Women with this phenotype show elevated inflammatory mediators, including TNF protein in menstrual discharge and IL-1β in endometrial tissue, together with dysregulation of NF-κB and COX pathway signaling [48,49,50,51,52]. Due to its incompletely understood pathogenesis, the origin of these molecular changes remains a significant focus of ongoing scientific investigation.

In the 2018 revision of the FIGO PALM-COEIN system, category N was renamed “not otherwise classified” to encompass a spectrum of potential causes that do not fit within the established categories and may or may not be identifiable by imaging or histopathology [40]. Earlier FIGO guidance noted that this category could include rare or insufficiently characterized entities, such as chronic endometritis, arteriovenous malformations, and myometrial hypertrophy, as well as disorders that may eventually be defined through biochemical or molecular methods [39].

We present CAM not as a replacement for other proposed explanations, but as an additional myometrium-centered hypothesis that may be relevant to a subset of women with AUB without an identified cause. Whether this proposed phenotype is associated with later fibroid development remains to be established in prospective studies. Thus, a subset of clinically unexplained AUB may reflect a chronic inflammatory uterine microenvironment rather than an isolated endometrial defect. All such cases are collectively grouped under abnormal uterine bleeding—not otherwise classified (AUB-N) in the popular FIGO-endorsed PALM COIEN classification [39]. Clinically, this is again the diagnosis of exclusion and most clinicians arrive ultimately at this AUB-N diagnosis when all other known causes are excluded and exhaustive imaging and laboratory tests all come back within normal limits [39,53]. Notably, the endometrial molecular markers of inflammation described above are examined in research settings and they are not part of routine standard of care evaluation of a reproductive-age adolescent or adult with regular heavy menstrual bleeding [54].

Recent reviews and mechanistic studies have highlighted potential links between chronic inflammation, uterine fibroids, and abnormal uterine bleeding. In uterine fibroids, immune-cell infiltration and upregulation of proinflammatory cytokines such as IL-1β, IL-6, TNF-α, and TGF-β have been reported in both fibroid tissue and adjacent endometrium, where they may impair endometrial receptivity and contribute to heavy menstrual bleeding [55]. Inflammation mediated by cytokines, chemokines, sex steroids, ECM remodeling, and immune cell infiltration is a shared mechanistic foundation of UFs, endometriosis, and adenomyosis [56]. Emerging evidence suggests that chronic inflammatory remodeling may link adenomyosis-like uterine injury with later fibroid development [56,57]. Inflammatory signaling pathways include NLRP3/NF-κB, Hippo/YAP, JAK/STAT, and Wnt/β-catenin [38,58]. Increased tissue stiffness may further contribute to the altered mechano-signaling and impaired myometrial contractility [37]. In parallel, chronic endometrial inflammation has been associated with nonstructural AUB in women without detectable structural pathology [59].

### 1.3. Chronic Aseptic Myometritis (CAM): A Hypothetical Framework Linking Myometrial Inflammation, AUB, and Fibroid Susceptibility

We speculate that many young women harboring pre-fibroid myometrium (MyoF) are asymptomatic or minimally symptomatic. However, in a subset with highly inflamed myometrium (severe MyoF), the diseased myometrium may exert a sustained microenvironmental field effect on the adjacent endometrium through paracrine cytokines, ROS, and extracellular vesicles (EVs)/exosomes carrying bioactive cargo such as microRNAs [1,55,56]. In this high-inflammation context, MyoF-derived EVs/exosomes can be taken up by endometrial endothelial and stromal cells and shift endometrial biology toward a pro-angiogenic, poorly stabilized vasculature. This reflects an imbalance of vascular endothelial growth factor (VEGF) and angiopoietin–Tie2 signaling and disruption in vasoconstrictor, vasodilator, and fibrinolytic balance that controls menstrual blood loss [48,55]. The fragile microvessels and a bleeding-prone endometrial phenotype is consistent with abnormal uterine bleeding in women without an identified structural lesion. Together, these observations suggest that MyoF is not merely a local phenomenon but a tissue-state capable of reprogramming endometrial biology. We therefore hypothesize that severe MyoF may represent an inflammatory and remodeling phenotype relevant to a subset of women with AUB without an identified cause. Consistent with this concept, our unpublished data shows that MyoF samples consistently failed to grow any live microbial cultures despite exhibiting marked inflammatory, oxidative stress, and ECM–remodeling transcriptomic signatures compared with matched MyoN tissue (unpublished data). Based on this evidence, we herein introduce the concept of Chronic Aseptic Myometritis (CAM), which is a proposed research construct for chronic, non-infectious inflammation of the myometrium.

Sterile inflammation is driven by endogenous danger molecules such as damage-associated molecular patterns (DAMPs), released during cell injury or stress in the absence of microbial stimuli [60]. Extracellular matrix (ECM) turnover, oxidative stress, and cellular injury that define the MyoF stage are also well-recognized sources of DAMPs/alarmins, including extracellular ATP, high-mobility group box 1 (HMGB1), mt DNA, and hyaluronan/fibronectin fragments [15,26,61]. These DAMPs engage pattern recognition receptors (PRRs), including Toll-like receptor 4 (TLR4) and the receptor for advanced glycation end products (RAGE), activating nuclear factor-κB (NF-κB) signaling and NLRP3 inflammasome (NLRP3–ASC–caspase-1), with subsequent IL-1β and IL-18 maturation [55,56,61]. This provides the mechanistic basis of aseptic MyoF tissue inflammation where inflammatory, oxidative, as well as ECM-remodeling signatures are perpetuated via self-derived danger signals rather than by infection. DAMP-driven inflammasome activation is self-amplifying in that it associates CAM to fibrosis and fibroid initiation. Sustained NLRP3 and NF-κB signaling drives macrophages recruitment and profibrotic TGF-β release, myofibroblast activation, and ECM deposition. This tissue remodeling generates more DAMPs, creating a feed-forward loop of chronic sterile inflammation and myometrial stiffening [14,62].

Beyond fibrosis, reactive oxygen species (ROS) and IL-1β enforce mutagenic pressure on resident myometrial stem cells, promoting oxidative DNA damage and impaired repair, leading to fibroid driver mutations [15,17]. Within the proposed framework, these pathways could link a MyoF-like inflammatory state with altered endometrial function and enhanced fibroid susceptibility; however, this sequence has not been demonstrated prospectively.

CAM may represent one possible biological explanation for AUB in a subset of women without an identified structural or systemic cause [40]. We further hypothesize that CAM contributes to vascular endothelial dysfunction, resulting in aberrant uterine bleeding patterns. If persistent and uncorrected, this chronic inflammatory myometrial milieu may also promote acquisition of fibroid driver mutations, ultimately predisposing to the emergence of fibroid lesions [20,23,57,63]. It is important to distinguish the elements of the CAM concept which are supported by existing data from those that remain hypothetical. Myometrial inflammation, TGF-β-related extracellular-matrix remodeling, oxidative stress with 8-OHdG accumulation, and their association with the fibroid biology are supported by experimental studies and in part human tissue studies [10,17,26,27,32,35]. However, most human data is based on cross-sectional comparisons of surgical samples and therefore establishes association rather than temporal sequence [27,31,32]. The concept that these findings integrate into a single clinical entity (CAM) that includes a subset of patients with AUB without an identified cause and enhances risk of fibroid development remains unproven and has not been demonstrated in prospective clinical studies. Herein, we present CAM as a hypothesis-generating framework for guiding future investigation rather than an established diagnosis.

In this review, CAM is proposed as a research framework for investigating whether chronic myometrial inflammation and remodeling contribute to the transition from normal myometrium (MyoN) to a MyoF-like state, altered endometrial function, AUB, and increased fibroid susceptibility in a subset of women. This review therefore examines whether the proposed CAM/MyoF framework may help identify early myometrial changes relevant to AUB and fibroid susceptibility.

### 1.4. Literature Search Strategy and Study Selection

This narrative review was informed by searches of PubMed and Google Scholar from database inception through 25 July 2026. Searches were conducted using combinations of terms related to the principal domains of the review, including “uterine fibroid,” “uterine leiomyoma,” “myometrium,” “myometrial inflammation,” “chronic aseptic myometritis,” “sterile inflammation,” “abnormal uterine bleeding,” “AUB-N,” “damage-associated molecular patterns,” “NLRP3 inflammasome,” “oxidative stress,” “oxidative DNA damage,” “MED12,” “extracellular matrix remodeling,” “TGF-β signaling,” “MyoN,” “MyoF,” “vitamin D,” and “epigallocatechin gallate.” Boolean operators were used to combine terms as appropriate for each topic.

Original epidemiological and clinical studies, human tissue studies, in vitro and in vivo mechanistic investigations, relevant clinical guidelines, and review articles published in English were considered. Titles and abstracts were initially evaluated for relevance to the proposed relationships among myometrial inflammation, abnormal uterine bleeding, fibroid initiation, and potential preventive interventions. Potentially relevant full-text publications were subsequently reviewed, and the reference lists of key articles were examined to identify additional pertinent studies. Publications were selected based on their relevance to the conceptual and mechanistic framework of the review. Because this was a narrative rather than a systematic review, study selection was not intended to be exhaustive, and no formal protocol registration, risk-of-bias assessment, or quantitative evidence synthesis was performed.

## 2. Gaps in Research and Clinical Implications

### Toward a Provisional Diagnostic Framework for CAM

As CAM is not a validated clinical diagnosis, the following framework is proposed only to guide research recruitment and biomarker development. Before CAM is considered, other causes of AUB should be assessed using the FIGO PALM-COEIN framework, together with evaluation for infectious or chronic endometritis and other relevant systemic conditions. CAM may be investigated as a possible non-infectious inflammatory and remodeling state of the myometrium in a subset of women with AUB without an identified cause.

A future research pathway would likely require a combination of clinical, biomarker, and imaging findings rather than a single test. Candidate measures include inflammatory cytokines and chemokines, angiogenic factors, extracellular-matrix and tissue-remodeling proteins, and oxidative-stress or DNA-damage markers measured in blood, urine, uterine fluid, or extracellular vesicles. Preliminary exploratory biomarker findings have been reported in conference proceedings but require validation in independent prospective cohorts [64]. Shear-wave elastography may also be investigated as a non-invasive measure of myometrial stiffness. A possible research pathway would involve confirming AUB, excluding established PALM-COEIN and infectious causes, assessing candidate biomarkers and myometrial stiffness, and following participants prospectively to determine whether the proposed phenotype predicts later fibroid development. None of these measures are currently validated for diagnosing CAM, and their thresholds, sensitivity, specificity, and reproducibility remain to be established.

## 3. CAM as a Potential Therapeutic Window for the Primary Prevention of Uterine Fibroids

### 3.1. Organoid Evidence for Reversal of the MyoF Phenotype

Emerging studies may benefit from investigating primary prevention strategies of uterine fibroids by targeting the underlying pathophysiological processes before tumor formation occurs. A recent organoid-based study [35] provides preliminary mechanistic evidence supporting the feasibility of investigating young women with AUB who may represent a CAM-associated pre-fibroid state. Using a three-dimensional (3D) organoid model derived from human myometrial stem cells (SCs), the study successfully recreated in vivo pathology of uterine fibroids. The organoids, composed of stem cells, smooth muscle, and fibroblasts, replicated key UF characteristics, like estrogen receptor (ERα) expression and hormonal responsiveness. The molecular analyses revealed significant ECM dysregulation within both fibroid UF and MyoF, as evidenced by elevated expression of COL1A1, COL3A1, and fibronectin compared to MyoN. ECM accumulation directly correlated with enhanced tissue stiffness; UF organoids showed nearly twofold higher elastic modulus relative to MyoN, confirming biomechanical transformation as a feature of early fibroid pathobiology. Notably, therapeutic exposure of MyoF organoids to vitamin D3 (100 nM), epigallocatechin gallate (EGCG, 10 µM), or their combination induced a significant decrease in stiffness, ECM marker expression and fibrotic pathways. The treated organoids exhibited molecular and mechanical properties like healthy MyoN tissue, indicating a phenotypic reversal from MyoF to MyoN. The finding proposes that these compounds not only suppress fibrogenic activity but may restore physiological myometrial architecture and function. Such results point toward a novel preventive paradigm whereby non-hormonal interventions could intercept early CAM-mediated fibrotic changes long before the development of fibroids.

### 3.2. Vitamin D and EGCG as Candidate Preventive Interventions

Clinically, this mechanistic insight may reshape how AUB is conceptualized in women without structural uterine lesions. Many such cases likely represent inflammatory and fibrogenic alterations within the myometrial tissue. Therefore, therapeutic intervention with vitamin D and EGCG represents a promising hypothesis that warrants further investigation. Based on available mechanistic and preclinical evidence, these compounds may have the potential to attenuate early myometrial remodeling and inflammation; however, prospective clinical studies are required to determine whether these biological effects translate into prevention of fibroid development. This approach would not only address immediate bleeding symptoms but targets disease at its molecular origin before irreversible tissue remodeling and tumorigenesis occur. Future clinical studies may determine whether these agents could eventually become mechanism-based preventive therapies.

In fact, even at the molecular level, vitamin D alone appears to play a significant part in myometrial health by modulating key pathways involved in fibrosis and inflammation. It exerts anti-inflammatory effects by modulating immune responses, reducing proinflammatory cytokine production, and regulating pathways central to fibroid development [65]. Specifically, vitamin D downregulates the expression of TGF-β and Wnt/β-catenin signaling, which are implicated in fibroid pathogenesis through their roles supporting cell proliferation and tumorigenesis [66]. Furthermore, vitamin D has been shown to reduce collagen and fibronectin production in fibroid cells, supporting its role in controlling fibrosis and tumor growth.

The cellular mechanism through which vitamin D exerts these antiproliferative effects was clarified by Sharan et al., who demonstrated that 1,25-dihydroxyvitamin D3 inhibits the proliferation of human uterine leiomyoma cells via catechol-O-methyltransferase (COMT), the same enzymatic axis implicated in EGCG’s mechanism of action, suggesting that vitamin D and EGCG may share converging molecular targets within leiomyoma cells [67]. Complementing this proliferative effect, Halder et al. showed that 1,25-dihydroxyvitamin D3 reduces TGF-β3-induced fibrosis-related gene expression in human uterine leiomyoma cells, directly attenuating the fibrotic gene program driven by one of the most potent profibrotic signals in the fibroid microenvironment [68]. Critically, these molecular effects translate to tumor-level efficacy in vivo: treatment with 1,25-dihydroxyvitamin D3 significantly shrank uterine leiomyoma tumors in the Eker rat model, with suppression of PCNA, cyclin D1, CDK1/2/4, BCL-2, and both estrogen and progesterone receptors, demonstrating that vitamin D can simultaneously target proliferative, antiapoptotic, and hormone-responsive pathways that sustain fibroid growth [69]. At the matrix level, Halder et al. further demonstrated that vitamin D treatment reduces extracellular matrix-associated protein expression in human uterine fibroid cells, confirming that its antifibrotic activity extends to ECM remodeling which is a key feature of both fibroid development and the MyoF phenotype [70]. It interferes with the activities of enzymes involved in ECM remodeling, such as matrix metalloproteinases (MMPs), contributing to the degradation and reconstruction of the ECM [65]. Together, these mechanistic and in vivo data establish vitamin D as a compound capable of simultaneously targeting proliferation, apoptosis resistance, fibrosis, and ECM remodeling in leiomyoma tissue [71].

In parallel, EGCG, the primary catechin in green tea, provides complementary therapeutic effects as a potent antioxidant and inhibitor of fibroid-promoting signaling networks. EGCG is a strong antioxidant that effectively scavenges reactive oxygen species (ROS) and activates antioxidant systems [72]. It has demonstrated efficacy in reducing fibroid size and symptoms in early clinical studies. For instance, a pilot randomized controlled clinical study demonstrated that EGCG (800 mg/day) reduced fibroid volume by 32.6% and symptom severity by 32.4%, with no observed adverse events [73].

The mechanistic basis for these effects is now well-established at the cellular and molecular level. Seminal work by Zhang et al. demonstrated that EGCG exerts direct antiproliferative and proapoptotic effects on human leiomyoma cells, downregulating proliferation markers PCNA and CDK4 alongside the anti-apoptotic protein BCL-2, while upregulating the proapoptotic effector BAX. These changes were accompanied by suppression of NF-κB-mediated survival signaling [74]. Such in vitro findings were subsequently validated in vivo where the green tea extract administered to nude mice bearing human leiomyoma xenografts produced significant tumor shrinkage, again with reductions in PCNA and CDK4, confirming that EGCG-driven antiproliferative activity is not an artifact of cell culture conditions [75]. Subsequent mechanistic work revealed that EGCG’s inhibition of leiomyoma cell proliferation is mediated, at least in part, through catechol-O-methyltransferase (COMT) [76]. More recently, Islam et al. extended this mechanistic landscape by demonstrating that EGCG targets the fibrotic signaling networks central to fibroid pathobiology specifically YAP, β-catenin, JNK, and AKT pathways, establishing EGCG as a multi-target inhibitor of the mechano-sensing and profibrotic cascades that drive ECM accumulation and fibroid growth [77]. In addition, studies indicate that EGCG suppresses the expression of key fibrotic mRNA or protein, including fibronectin (FN1), collagen type I (COL1A1), plasminogen activator inhibitor-1 (PAI-1), connective tissue growth factor (CTGF), and actin alpha 2, smooth muscle (ACTA2) in fibroid cells, thereby limiting ECM accumulation [64]. Ali et al. demonstrated that in human uterine fibroid cells, the combination of 1,25-dihydroxyvitamin D3 and ulipristal acetate exerted a substantially stronger antifibrotic effect than ulipristal acetate alone [78]. The combined treatment significantly reduced the expression of key extracellular matrix proteins, including collagen type I and fibronectin, and suppressed the profibrotic cytokine TGF-β3. Immunofluorescence analysis further demonstrated a marked reduction in the dense, disorganized collagen fiber network characteristic of fibrotic tissue. In addition, the combination downregulated multiple fibrosis-associated genes, including COL1A, FN1, TGF-β3, fibromodulin, biglycan, and versican. These coordinated decreases in ECM production and profibrotic signaling are consistent with reversal of a myofibroblast-like fibrotic phenotype and restoration toward a less activated cellular state [78].

**Figure 2:**
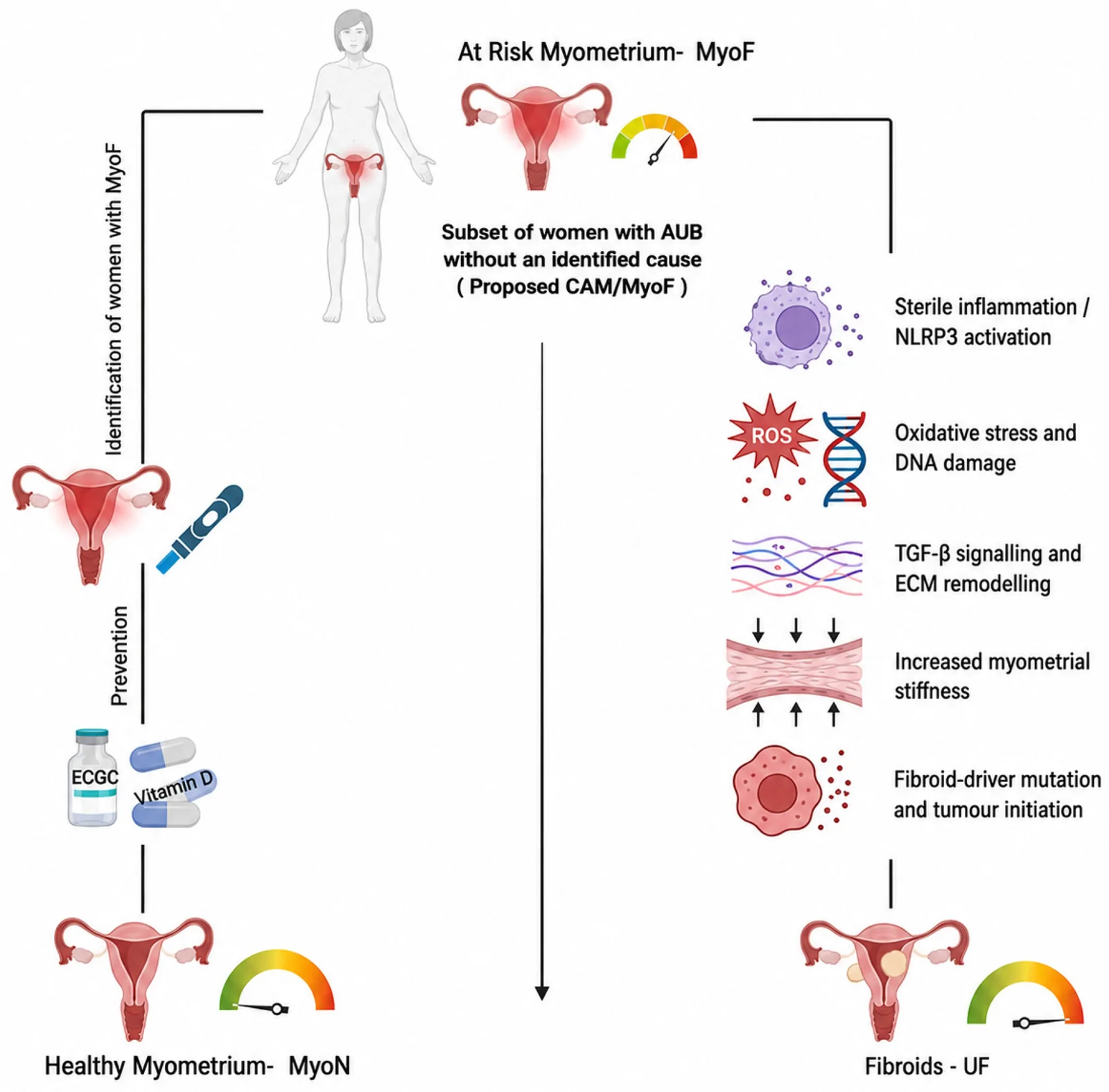
Proposed research framework for CAM/MyoF and possible progression to uterine fibroids. The schematic illustrates a hypothesis-generating model in which a subset of women with AUB without an identified cause may exhibit a proposed CAM/MyoF phenotype. The right-hand pathway shows proposed mechanisms that may contribute to fibroid development, while the left-hand pathway depicts investigational identification and potential non-hormonal interventions, including vitamin D and EGCG. These pathways remain unvalidated and require prospective clinical investigation. Figure created with https://www.biorender.com/.

These effects further strengthen the role of both vitamin D and EGCG as potential strategies to reverse early CAM or prevent fibroid development, aligning with the Evidence-Based Approach for Secondary Prevention (ESCAPE) of UF management [64]. The ESCAPE approach advocates for the use of vitamin D (4000 IU/day) and EGCG (800 mg/day). The ESCAPE framework has proposed vitamin D and EGCG as promising candidate interventions based on existing mechanistic and early clinical evidence. However, their role in primary prevention remains investigational and requires confirmation in well-designed prospective clinical trials (Figure 2).

If future translational and clinical studies validate CAM as a distinct clinicopathologic entity, consideration could eventually be given to establishing a dedicated diagnostic classification. CAM may represent a research phenotype for investigating myometrial inflammation and remodeling in a subset of women with AUB without an identified cause [39], particularly in those who may reflect a pre-fibroid state. A potential investigational, non-hormonal approach would be to use decaffeinated EGCG (800 mg/day, administered with food) together with vitamin D supplementation (4000 IU/day) to reduce inflammation, reverse early myometrial remodeling, and lower fibroid risk in patients with suspected CAM. This approach may also help relieve symptoms in symptomatic patients, as not all individuals with CAM are expected to be asymptomatic. More severe forms of CAM may present with dysmenorrhea, heavy menstrual bleeding, or other abnormal uterine bleeding despite a negative standard clinical workup.

## 4. Research Gaps in Clinical Practice and Future Directions

### 4.1. Limitations of the Proposed CAM Framework

While the CAM hypothesis integrates emerging mechanistic and epidemiologic observations into a unified conceptual model, several important limitations should be acknowledged. First, CAM is currently a hypothesis-generating construct rather than a validated clinical diagnosis, and no validated biomarkers, imaging criteria, or standardized diagnostic tests currently exist. Second, most supporting evidence derives from mechanistic studies, cross-sectional tissue analyses, organoid models, and observational clinical studies, which cannot establish temporal or causal relationships. Finally, whether CAM represents a distinct clinicopathologic entity, predicts future fibroid development, or identifies women who may benefit from preventive interventions remains unknown. Prospective longitudinal cohorts, translational biomarker studies, and randomized clinical trials will be required before this framework can be translated into routine clinical practice. Accordingly, the purpose of this review is not to propose immediate changes to patient management but rather to highlight an important, understudied area of women’s health and stimulate future investigation.

### 4.2. Future Research Directions

Despite advances in understanding the biological features of MyoF, reliable methods for identifying this proposed state before fibroid development are lacking. Two complementary investigational approaches may help address this gap. First, MyoF-related liquid biomarkers, including proteins, microRNAs, and extracellular vesicles, could be investigated in organoid-conditioned media, blood, and urine. Second, SWE may provide a measure of myometrial stiffness, a feature of the MyoF state. Preliminary studies suggest that transvaginal SWE can quantify myometrial stiffness and may detect differences between selected study groups [79]. However, its validity for identifying a MyoF-like phenotype or predicting fibroid development has not been established. Until prospectively validated, SWE should remain an investigational research measure [79]. Including SWE into routine gynecologic ultrasound could identify women with increased myometrial stiffness and may benefit from early intervention. Until these approaches are prospectively validated, they should remain investigational. Prospective studies are needed to validate candidate biomarkers and imaging-based endpoints for MyoF detection.

A separate research priority is to determine whether candidate non-hormonal interventions can safely improve fibroid-related clinical or biological outcomes. In this regard, the ongoing Fibroid Research Investigating EGCG Natural Compound in Green Tea (FRIEND) trial represents a pivotal step toward prospective clinical evaluation of EGCG, as a non-hormonal therapeutic strategy for uterine fibroids. Although its primary endpoints might not be mechanistic, its outcomes will provide clinical-grade evidence on whether EGCG-based intervention can improve reproductive outcomes and modify fibroid-associated disease activity, such as fibroid volume and symptom burden [80]. FATIMA trial addresses the prevention paradigm and evaluates whether combined vitamin D3 and EGCG supplementation can reduce fibroid recurrence following myomectomy (ClinicalTrials.gov, NCT07647198). Future translational sub-studies including myometrial stiffness measurements by SWE and circulating biomarker(s) profiling could help determine whether clinical responses to EGCG are associated with anti-inflammatory and anti-fibrotic remodeling pathways, including the MyoF-to-MyoN reversal mechanism. Vitamin D and EGCG may be considered candidate interventions for future study, but current evidence is insufficient to support their use as first-line primary prevention.

## 5. Conclusions

CAM is proposed as a hypothesis-generating framework that may help explain a subset of abnormal uterine bleeding without an identified cause. By integrating emerging evidence on myometrial sterile inflammation, extracellular-matrix remodeling, oxidative DNA damage, and fibroid biology, the framework identifies testable hypotheses regarding the transition from normal myometrium to a pre-fibroid MyoF-like state and its possible contribution to fibroid susceptibility. Importantly, CAM has not been validated as a clinical diagnosis; its proposed diagnostic criteria are provisional, and the relationships among myometrial inflammation, AUB, and fibroid development remain to be established. Prospective longitudinal and further mechanistic studies are needed to validate candidate biomarkers, establish temporal and causal relationships, and determine whether targeted, non-hormonal interventions can prevent progression to clinically apparent fibroids. Combining early detection with targeted intervention will contribute to providing a promising framework for primary prevention of uterine fibroids and better care for women with higher risk.

## Data Availability

No new data were created or analyzed in this study. Data sharing is not applicable to this article.

## Abbreviations

The following abbreviations are used in this manuscript:

3D	Three-dimensional
8-OHdG	8-hydroxy-2′-deoxyguanosine
ACTA2	Actin alpha 2, smooth muscle
AUB	Abnormal uterine bleeding
AUB-E	AUB due to endometrial causes
AUB-N	AUB, not otherwise classified
AUB-O	AUB due to ovulatory dysfunction
BMI	Body mass index
CAM	Chronic aseptic myometritis
CD44	Cluster of differentiation 44
CI	Confidence interval
COL1A1	Collagen type I alpha 1
COL3A1	Collagen type III alpha 1
COX	Cyclooxygenase
CRP	C-reactive protein
CTGF	Connective tissue growth factor
DNA	Deoxyribonucleic acid
DUB	Dysfunctional uterine bleeding
ECM	Extracellular matrix
EGCG	Epigallocatechin gallate
EGF	Epidermal growth factor
ER	Estrogen receptor
ERα	Estrogen receptor alpha
ESCAPE	Evidence-Based Approach for Secondary Prevention (of UF)
EVs	Extracellular vesicles
FDA	(U.S.) Food and Drug Administration
FH	Fumarate hydratase
FIGO	International Federation of Gynecology and Obstetrics
FN1	Fibronectin 1
FORT	Free Oxygen Radical Test
GPCR(s)	G protein-coupled receptor(s)
GPER	G protein-coupled estrogen receptor
HMGA2	High mobility group AT-hook 2
hs-CRP	High-sensitivity C-reactive protein
ICAM-1	Intercellular adhesion molecule 1
IFN-γ	Interferon gamma
IL-1β	Interleukin 1 beta
IL-6	Interleukin 6
JAK/STAT	Janus kinase/signal transducer and activator of transcription
MAPK	Mitogen-activated protein kinase
MCP-1	Monocyte chemoattractant protein 1
MED12	Mediator complex subunit 12
MMP(s)	Matrix metalloproteinase(s)
mRNA	Messenger ribonucleic acid
MyoF	Pre-fibroid myometrium/myometrium adjacent to fibroids
MyoN	Normal (fibroid-free) myometrium
NF-κB	Nuclear factor kappa B
NICE	National Institute for Health and Care Excellence
NLRP3	NLR family pyrin domain containing 3
OR	Odds ratio
PAI-1	Plasminogen activator inhibitor-1
PALM-COEIN	Polyp, Adenomyosis, Leiomyoma, Malignancy & hyperplasia; Coagulopathy, Ovulatory dysfunction, Endometrial, Iatrogenic, Not otherwise classified
PCOS	Polycystic ovary syndrome
RAD50/RAD51	DNA repair proteins RAD50/RAD51
ROS	Reactive oxygen species
RR	Relative risk
SC(s)	Stem cell(s)
Stro-1	Stromal cell antigen 1
SWE	Shear wave elastography
TGF-β	Transforming growth factor beta
TNF-α	Tumor necrosis factor alpha
UF(s)	Uterine fibroid(s)
USD	United States dollar
YAP	Yes-associated protein
γH2AX	Phosphorylated gamma H2A histone family member X
